# Plasma Exosomes Derived From Patients With End-Stage Renal Disease and Renal Transplant Recipients Have Different Effects on Vascular Calcification

**DOI:** 10.3389/fcell.2020.618228

**Published:** 2021-01-28

**Authors:** Xiao Lin, Ting Zhu, Feng Xu, Jia-Yu Zhong, Fuxingzi Li, Su-Kang Shan, Feng Wu, Bei Guo, Ming-Hui Zheng, Yi Wang, Qiu-Shuang Xu, Xiao-Bo Liao, Hong-Yu Lu, Xu-Biao Xie, Ling-Qing Yuan

**Affiliations:** ^1^Department of Radiology, The Second Xiangya Hospital, Central South University, Changsha, China; ^2^Department of Endocrinology and Metabolism, National Clinical Research Center for Metabolic Diseases, The Second Xiangya Hospital, Central South University, Changsha, China; ^3^Department of Endocrinology, Central Hospital of Yiyang, Yiyang, China; ^4^Department of Geriatrics, The Second Xiangya Hospital, Central South University, Changsha, China; ^5^Department of Pathology, The Second Xiangya Hospital, Central South University, Changsha, China; ^6^Department of Cardiovascular Surgery, The Second Xiangya Hospital, Central South University, Changsha, China; ^7^Xiangya Medical College, Central South University, Changsha, China; ^8^Department of Kidney Transplantation, The Second Xiangya Hospital, Central South University, Changsha, China

**Keywords:** ESRD, exosomes, vascular calcification, Fetuin-A, MGP, Annexin-A2

## Abstract

End-stage renal disease (ESRD) patients usually develop extensive and progressive vascular calcification, and lots of calcification inhibitors as well as procalcifying factors are involved in the process. However, the mechanisms of vascular calcification in ESRD patients are still ill-defined. In the present study, we found that the plasma exosomes derived from ESRD patients (ESRD-Ex) promoted calcification of vascular smooth muscle cells (VSMCs) significantly, while plasma exosomes from renal transplant recipients (RTR-Ex) could partially attenuate VSMCs calcification. Moreover, the protein concentration of ESRD-Ex was significantly higher than plasma exosomes from the normal health control group (Nor-Ex) and RTR-Ex, and the content of both matrix gla protein (MGP) and Fetuin-A, the calcification inhibitors, were prominently lower in ESRD-Ex than those in Nor-Ex. The content of Annexin-A2, one of the calcification promoters, was significantly higher in ESRD-Ex and RTR-Ex than that in Nor-Ex. However, bone morphogenetic protein (BMP-2) and receptor activator for nuclear factor-κB ligand (Rankl) had no significant difference among the three groups. In addition, the content of Fetuin-A in RTR-Ex was higher than that in ESRD-Ex, although it was still lower than that in Nor-Ex. Furthermore, the levels of both Fetuin-A and MGP in plasma exosomes were negatively while the levels of Annexin-A2 in plasma exosomes was positively correlated to coronary artery calcification scores (CACS). These results indicated that ESRD-Ex significantly promoted VSMCs calcification, while renal transplantation could partially attenuate the procalcification effect of exosomes. Fetuin-A and MGP were decreased, but Annexin-A2 was increased in ESRD-Ex, and renal transplantation could increase the level of Fetuin-A rather than MGP.

## Introduction

Vascular calcification is mainly characterized as the medial of aortic calcification (termed as Mönckeberg's calcification), and it often happens in patients with chronic kidney disease (CKD), especially end-stage renal disease (ESRD) (Nitta and Ogawa, 2015; Rangaswami et al., 2019). Lots of studies have demonstrated that vascular calcification is not a simple passive process of calcium and phosphorus deposition but an active regulatory process, and the transdifferentiation of vascular smooth muscle cells (VSMCs) into osteoblast-like cells is regarded to be the key pathophysiological factor of vascular calcification (Peng et al., 2017; Lin et al., 2018, 2019c). However, the specific mechanisms of vascular calcification in patients with ESRD are still not entirely elucidated.

Exosomes (Ex) are of endosomal origin from multivesicular bodies. They have a diameter of 30–100 nm, and they are released into the extracellular matrix. Numerous studies have reported that exosomes are usually regarded as mediator of cell-to-cell communication in physiological and pathological conditions because of their variety and the abundance of specific cargos, including proteins, lipids, and nucleic acids (Zhang et al., 2018; Li F. X. et al., 2019; Wu et al., 2019). Recently, increasing evidence have shown that exosomes also played an important role in regulating vascular calcification (Lin et al., 2019b; Li S. et al., 2019). For instance, Li et al. reported that exosomes derived from high-glucose-induced endothelial cells could carry versican protein to accelerate VSMCs calcification (Lin et al., 2019c). Our previous study demonstrated that melatonin alleviated vascular calcification through an exosomal miR-204/miR-211 cluster in a paracrine manner (Xu et al., 2020). However, the role and mechanisms of exosomes in regulating vascular calcification in patients with ESRD is not very clear.

Vascular calcification is an active regulatory process, and a lot of calcification inhibitors as well as procalcifying factors are involved (Leopold, 2015; Back et al., 2018). Numerous studies have confirmed that Fetuin-A and matrix gla protein (MGP) are well-known calcification inhibitors, and some procalcifying factors, including bone morphogenetic protein (BMP-2), receptor activator of nuclear factor-κB ligand (Rankl), and Annexin-A2, are involved in regulating vascular calcification (Chen et al., 2008; Leopold, 2015; Viegas et al., 2015; Back et al., 2018). When BMP-2 was increased in uremic serum, it could enhance VSMCs calcification *in vitro* (Chen et al., 2006), and Rankl promoted osteoblastic differentiation of VSMCs by promoting endothelial to release BMP-2 (Davenport et al., 2016). In addition, studies demonstrated that Annexin-A2 was significantly increased in calcified bovine VSMCs (Chen et al., 2008), and inhibition of annexin activity decreased chondrocyte mineralization (Kapustin et al., 2011). Herein, it is urgent to investigate whether the calcification-related factors are involved in exosomes regulating vascular calcification in patients with ESRD.

In the present study, we found that plasma exosomes derived from patients with ESRD (ESRD-Ex) promoted VSMCs calcification, while plasma exosomes from renal transplantation patients (RTR-Ex) could partially attenuate VSMCs calcification. The mechanism study demonstrated that the content of calcification inhibitors (Fetuin-A and MGP) decreased significantly, while Annexin-A2, one of procalcifying factors, increased greatly in both plasma and plasma exosomes derived from patients with ESRD. On the other hand, renal transplantation could partially restore the level of Fetuin-A in plasma exosomes and then attenuate vascular calcification.

## Results

### Vascular Calcification Is Associated With Calcium and Phosphorus Metabolism Disorders in Patients With ESRD

Twenty-four participants were involved in this study, and the age of the participants ranged from 25 to 56 years old. The course of the ESRD (starting from the time of diagnosis of ESRD) group ranged from 0.42 to 6 years, with an average course of 3.12 years. These patients included two patients with peritoneal dialysis and six patients with hemodialysis. The course of the RTR (starting from the time of renal transplantation for the ESRD group ranged from 0.33 to 5.5 years, with an average course of 1.9 years). Among them, three patients underwent peritoneal dialysis and five patients underwent hemodialysis before renal transplantation. The clinical characteristics of the participants are shown in Table 1.

**Table 1:** Biochemical characteristics in normal health, ESRD, and RTR.

	**Normal**	**ESRD**	**RTR**
Age (years)	37.75 ± 4.333	33.13 ± 1.42	39.88 ± 2.844
Cre (μmol/L)	61.56 ± 3.327	1284 ± 143.2^*^	117.1 ± 15.99^*^^#^
BUN (mmol/L)	4.763 ± 0.5495	29.4 ± 3.669^*^	7.598 ± 0.9367^*^^#^
UA (μmol/L)	307.7 ± 20.34	438.1 ± 25.65^*^	375.1 ± 32.18^*^^#^
csCa (mg/dl)	8.753 ± 0.1139	9.575 ± 0.3389^*^	9.176 ± 0.1603
P (mg/dl)	3.042 ± 0.1964	6.312 ± 0.8433^*^	2.767 ± 0.2039^#^
Ca × P (mg^2^/dl^2^)	26.58 ± 1.624	59.95 ± 7.89^*^	25.49 ± 2.1^#^
PTH (mmol/l)	4.999 ± 0.4806	61.26 ± 10.89^*^	9.089 ± 1.015^#^
TG (mmol/l)	1.474 ± 0.2042	2.368 ± 0.6717	1.425 ± 0.1632
TC (mmol/l)	3.363 ± 0.275	4.276 ± 0.4811	4.12 ± 0.3546
HDL (mmol/l)	0.905 ± 0.08356	1.075 ± 0.0964	1.289 ± 0.1698
LDL (mmol/l)	2.099 ± 0.2188	2.581 ± 0.4126	2.393 ± 0.1939
25(OH)D (nmol/l)	50.63 ± 5.018	35.5 ± 3.157^*^	40.88 ± 3.108

*Cre, creatinine; BUN, blood urea nitrogen; UA, uric acid; csCa, corrected serum calcium; P, phosphorus; Ca × P, calcium–phosphorus product; PTH, parathyroid hormone; TG, triglyceride; TC, total cholesterol; HDL, high-density lipoprotein; LDL, low-density lipoprotein*.

* *p < 0.05, compared with normal health control group*.

# *p < 0.05, compared with ESRD group*.

The results showed that there were no significant differences in the distribution of age, triglyceride (TG), total cholesterol (TC), high-density lipoprotein (HDL), or low-density lipoprotein (LDL) among the three groups. However, the serum creatinine (Cre), uric acid (UA), and blood urea nitrogen (BUN) had statistically significant differences among the three groups. Compared with the normal health control, the levels of corrected serum calcium (csCa), phosphorus (P), calcium–phosphorus product (Ca × P), and parathyroid hormone (PTH) in patients with ESRD were significantly increased. The levels of P, Ca × P, and PTH decreased significantly in the RTR group when compared with the ESRD group. However, there was no significant difference in csCa between ESRD and RTR groups. Besides, the level of 25(OH)D in patients with ESRD was much lower than that in normal healthy control, and it was a slightly increased in patients with RTR, but there was no significant difference between the ESRD and RTR group. These results suggested that vascular calcification might be related to high levels of Ca, P, and PTH and low levels of 25(OH)D in patients with ESRD, and renal transplantation may attenuate vascular calcification via decreasing the levels of P and PTH rather than Ca and 25(OH)D.

### Identification of Exosomes

**Figure 1:**
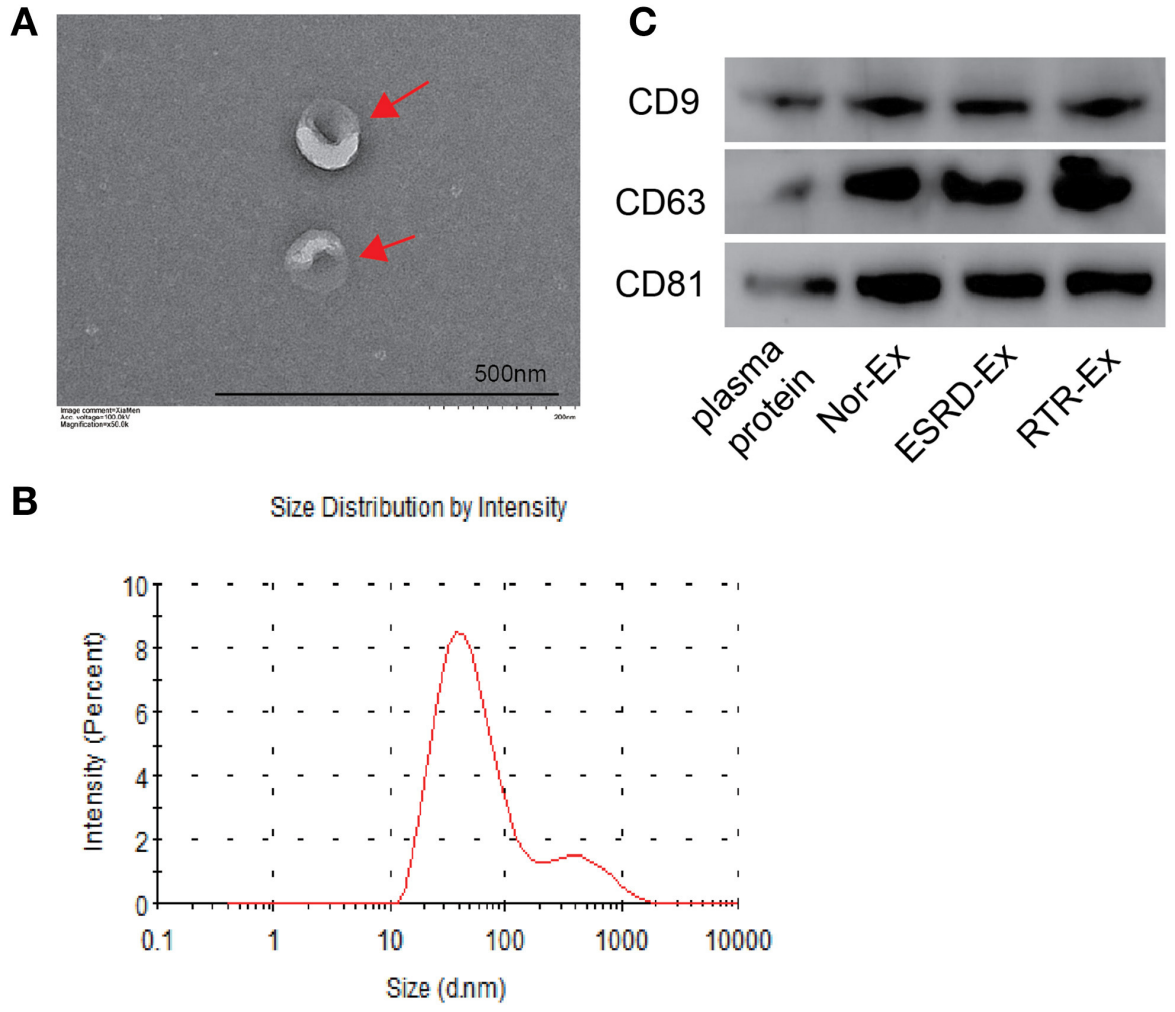
The characteristics of exosomes. **(A)** The morphology of exosomes was observed by TEM. The arrows indicated the exosomes. The scale bar was 500 nm. *n* = 3. **(B)** The diameter distribution of exosomes was measured by a molecular size analyzer. **(C)** Exosomes markers CD9, CD63, and CD81 were detected by western blot. The representative images were shown. TEM: transmission electron microscope; Nor-Ex, the plasma exosomes derived from normal health control; ESRD-Ex, the plasma exosomes derived from ESRD patients; RTR-Ex, the plasma exosomes derived from renal transplant recipients.

Exosomes were isolated from the plasma of normal health control (Nor-Ex), ESRD patients (ESRD-Ex), and RTR patients (RTR-Ex). The transmission electron microscope (TEM) showed that the exosomes displayed a bilayer structure morphology, and the mean diameter detected by a molecular size analyzer was 85.5 ± 3.96 nm (Figures 1A,B). Western blot analysis further verified that the presence of the exosomes markers, including CD9, CD63, and CD81, were increased in Ex when compared with total plasma protein (Figure 1C). All of these features confirmed that the vesicles were actually exosomes.

### ESRD-Ex Promoted VSMCs Calcification, While RTR-Ex Inhibited VSMCs Calcification

**Figure 2:**
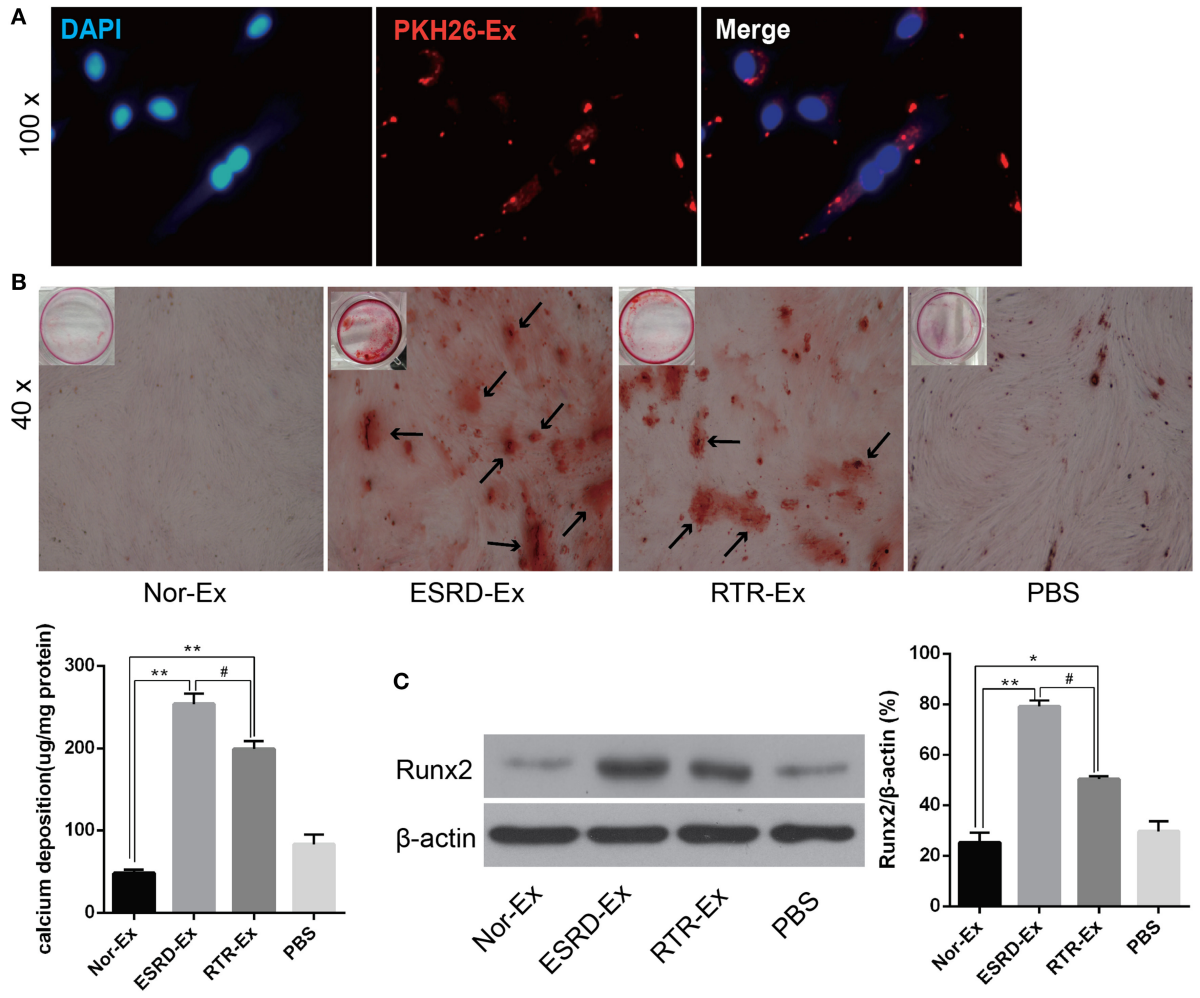
ESRD-Ex could be uptaken by VSMCs and promoted VSMCs calcification. **(A)** Fluorescence microscopy analysis revealed that PKH26-Ex could be uptaken and incorporated into VSMCs. **(B)** Alizarin Red S staining showed the mineralized nodules in VSMCs treated with different kinds of exosomes, and the calcium contents were quantified by spectrophotometry. The arrows indicated the mineralized nodules. **(C)** The expression of Runx2 was measured by western blot in VSMCs treated with different kinds of exosomes. The representative images were shown. *n* = 3. **p < 0.01, *p < 0.05, compared with Nor-Ex. ^#^*p* < 0.05, compared with ESRD-Ex. Nor-Ex, the plasma exosomes derived from normal health control; ESRD-Ex, the plasma exosomes derived from ESRD patients; RTR-Ex, the plasma exosomes derived from renal transplant recipients.

To explore the mechanisms involved in the vascular calcification, Nor-Ex, ESRD-Ex, and RTR-Ex were used to treat VSMCs to induce VSMCs calcification. Firstly, the fluorescence microscopy analysis revealed that PKH26-labeled exosomes (PKH26-Ex) could be taken up and incorporated into VSMCs (Figure 2A). The Alizarin Red S staining showed that both ESRD-Ex and RTR-Ex could enhance VSMCs calcification by increasing mineralized nodules formation and the level of Runx2 protein, while Nor-Ex could inhibit VSMCs calcification. However, mineralized nodules formation and the level of Runx2 protein were decreased in VSMCs treated with RTR-Exo than that with ESRD-Ex (Figures 2B,C). These data suggested that ESRD-Ex promoted VSMCs calcification significantly, while renal transplantation could partially attenuate the procalcification effect of exosomes.

### The Content of Calcification Inhibitors Were Decreased in ESRD-Ex

**Figure 3:**
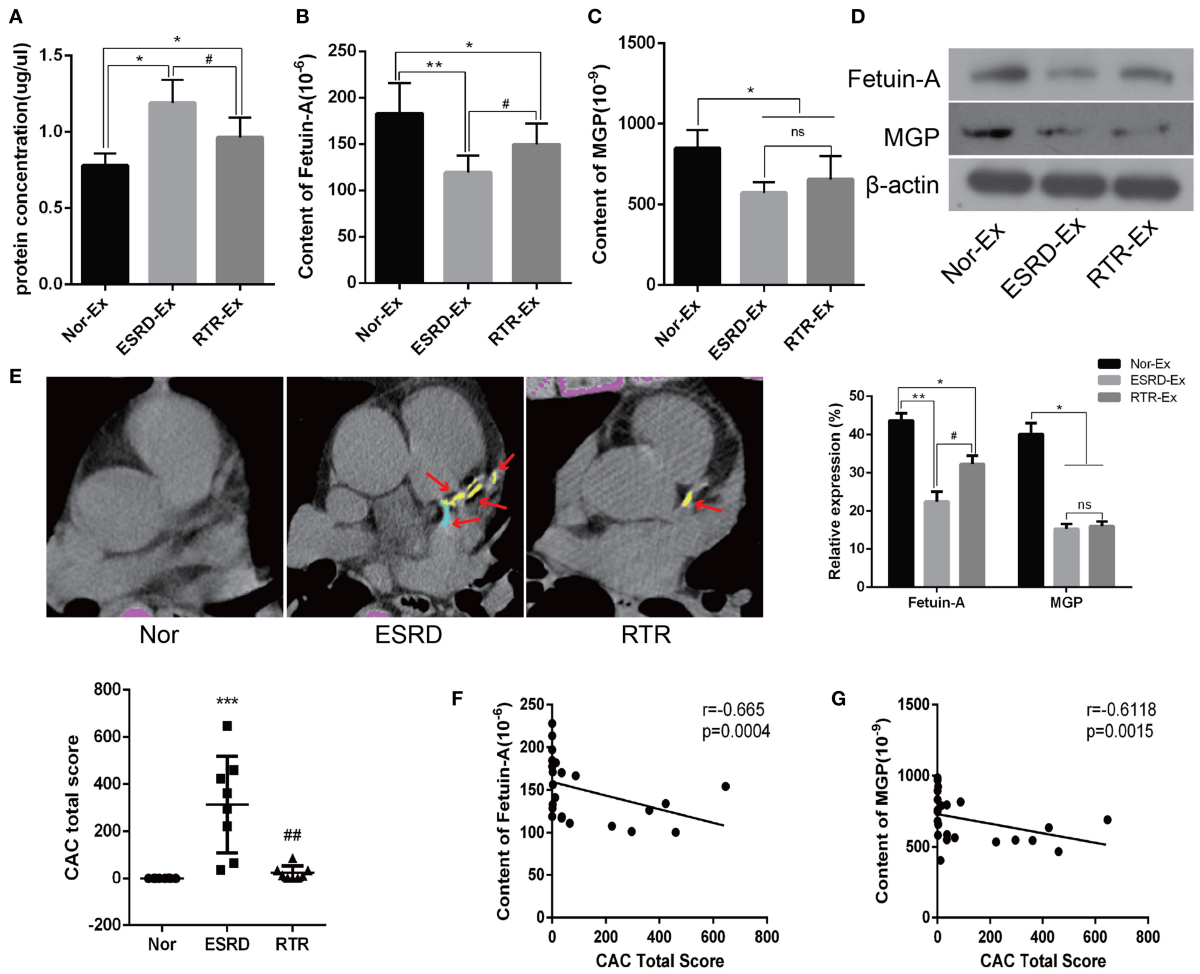
Fetuin-A and MGP were decreased in ESRD-Ex. **(A)** The protein concentration of exosomes in Nor-Ex, ESRD-Ex, and RTR-Ex. **(B,C)** The content of Fetuin-A and MGP in Nor-Ex, ESRD-Ex, and RTR-Ex were measured by ELISA. **(D)** Western blot analyses detected the levels of Fetuin-A and MGP protein. *n* = 3. **(E)** The CAC total score of Nor, ESRD, and RTR patients were calculated by a multilayer spiral CT. *n* = 8. The representative images were shown. The arrows indicated the calcified part of different branches of the coronary artery. **(F,G)** The spearman analysis showed the correlations between the content of plasma exosomes Fetuin-A and MGP with CAC total score, respectively. *n* = 24. ***p < 0.001, **p < 0.01, *p < 0.05, compared with Nor. ^##^*p* < 0.01, ^#^*p* < 0.05, compared with ESRD. ns, not significant; Nor-Ex, the plasma exosomes derived from normal health control; ESRD-Ex, the plasma exosomes derived from ESRD patients; RTR-Ex, the plasma exosomes derived from renal transplant recipients; CAC, coronary artery calcification.

To further explore the mechanisms of ESRD-Ex regulating VSMCs calcification, we determined the calcification promoters and inhibitors in Nor-Ex, ESRD-Ex, and RTR-Ex. Previous studies had demonstrated that Fetuin-A and MGP were calcification inhibitors (Marechal et al., 2011; Viegas et al., 2015; Roumeliotis et al., 2020). Firstly, we found that both the plasma concentration of Fetuin-A and MGP were decreased significantly in ESRD patients compared with normal health control, while RTR could increase, to some extent, the plasma concentration of Fetuin-A and MGP (Supplementary Figures 1A,B). Meantime, the protein concentration of ESRD-Ex and RTR-Ex were much higher than that of Nor-Ex, although the protein concentration of RTR-Ex was lower than that of ESRD-Ex (Figure 3A). The ELISA results further showed that the content of Fetuin-A was the highest in Nor-Ex and decreased significantly in ESRD-Ex. The content of Fetuin-A was increased in RTR-Ex, although it remained lower than that in Nor-Ex (Figure 3B). Besides, the content of MGP in both ESRD-Ex and RTR-Ex was lower than that in Nor-Ex, but the levels of MGP between ESRD-Ex and RTR-Ex had no significant difference (Figure 3C). In addition, western blot analyses verified the similar results as the ELISA (Figure 3D). Coronary artery calcification (CAC) is an important form of vascular calcification, and the CAC total score (CACS) is often used to evaluate the severity of vascular calcification (Greenland et al., 2018; Sakaguchi et al., 2019). CACS showed that the mean CACS in ESRD patients was 313.9 ± 204.98, and it was reduced significantly in patients with RTR (Figure 3E). Furthermore, the spearman analyses showed that the content of both Fetuin-A and MGP in plasma exosomes was negatively correlated to CACS (Figures 3F,G). These data demonstrated that Fetuin-A and MGP were decreased in ESRD-Ex, and renal transplantation could partially increase the level of Fetuin-A rather than MGP in the exosomes.

### Calcification Promoter Annexin-A2 Was Increased in ESRD-Ex

**Figure 4:**
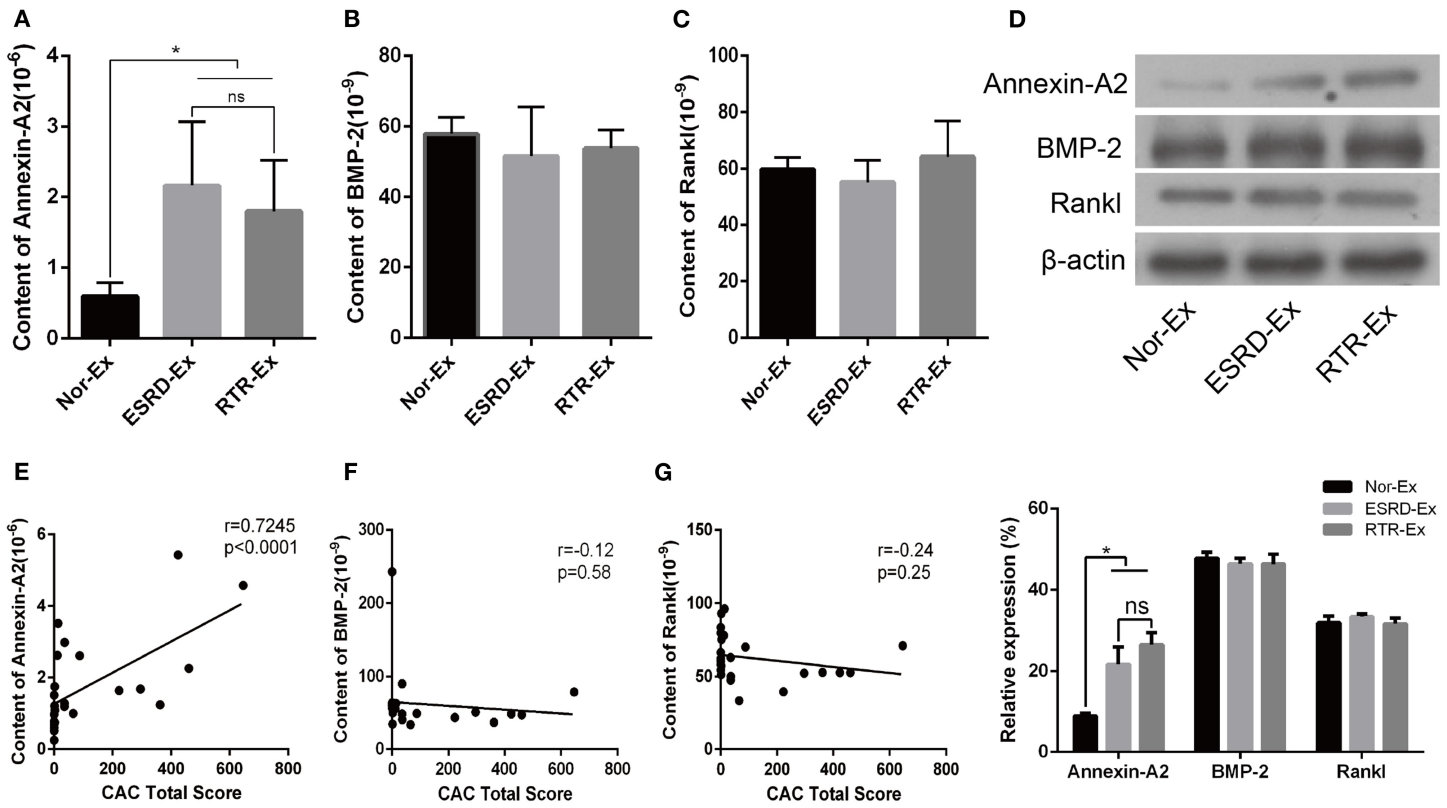
Annexin-A2 increased in ESRD-Ex. **(A–C)** The content of Annexin-A2, BMP-2, and Rankl in Nor-Ex, ESRD-Ex, and RTR-Ex were measured by ELISA. **(D)** Western blot detected the protein levels of Annexin-A2, BMP-2, and Rankl. *n* = 3. **(E–G)** The spearman analysis showed the correlations between the content of plasma exosomes Annexin-A2, BMP-2, and Rankl with CAC total score, respectively. *n* = 24. *p < 0.05, compared with Nor-Ex. ns, not significant; CAC, coronary artery calcification; Nor-Ex, the plasma exosomes derived from normal health control; ESRD-Ex, the plasma exosomes derived from ESRD patients; RTR-Ex, the plasma exosomes derived from renal transplant recipients.

Calcification promoters are another kind of important factor that could affect the progress of vascular calcification, and previous studies showed that Annexin-A2, BMP-2, and Rankl were calcification promoters (Chen et al., 2008; Osako et al., 2010; Davenport et al., 2016; Roumeliotis et al., 2020). In the present study, we found that the plasma concentration of Annexin-A2 and BMP-2 were increased significantly in patients with ESRD when compared with normal health control. However, the plasma concentration of Rankl was a little lower in patients with ESRD, but there was no significant difference among the three groups (Supplementary Figures 1C–E). Meantime, the content of Annexin-A2 in both ESRD-Ex and RTR-Ex was higher than that in Nor-Ex, but there was no significant difference between ESRD-Ex and RTR-Ex (Figure 4A). Nevertheless, the content of both BMP-2 and Rankl had no significant difference among the three groups (Figures 4B,C). Besides, western blot analyses showed the results similar to the ELISA tests (Figure 4D). Meanwhile, the content of Annexin-A2 in plasma exosomes was positively related to CACS (Figure 4E), while that of BMP-2 and Rankl had no significant correlation with CACS (Figures 4F,G). These results demonstrated that it was Annexin-A2, rather than BMP-2 and Rankl, increased in ESRD-Ex, and renal transplantation had no significant effect on the contents of Annexin-A2, BMP-2, and Rankl.

## Discussion

In the present study, we clarified that ESRD-Ex promoted VSMCs calcification significantly, while renal transplantation could partially attenuate the procalcification effect of exosomes. The mechanism study found that Fetuin-A and MGP were decreased, but Annexin-A2 was increased in ESRD-Ex. Moreover, renal transplantation could increase the level of Fetuin-A rather than MGP in plasma exosomes, but that of Annexin-A2, BMP-2, and Rankl was not affected. In addition, we found that the content of plasma exosomes Fetuin-A and MGP was negatively while that of Annexin-A2 was positively correlated to CACS.

Vascular calcification is common, and the related cardiovascular complications are the main causes of mortality in patients with ESRD. Renal replacement therapy including hemodialysis, peritoneal dialysis, and renal transplantation are the main treatments for patients with ESRD (Rangaswami et al., 2019; Chen et al., 2020). Vascular medial calcification is mainly caused by the osteoblastic transdifferentiation of VSMCs, and disorder of calcium and phosphate metabolism is the main pathogenesis of vascular medial calcification (Lin et al., 2019c; Xu et al., 2019). In this study, blood calcium usually refers to the calcium ion in plasma, and blood phosphorus refers to the plasma inorganic phosphorus. csCa was used in our study because serum Ca was affected by the concentration of serum albumin. Under physiological conditions, the concentrations of serum Ca and P are kept in balance, and the Ca × P is usually used as an indicator of osteogenesis (Viegas et al., 2018). In our present study, the csCa, P, and Ca × P were significantly higher in ESRD patients. Although there is no significant difference in the csCa between ESRD and RTR patients, the P and Ca × P were deceased greatly in RTR patients and had no significant difference when compared with that in the normal health control. These data suggested that Ca and P metabolism disorder is one of the mechanisms to promote vascular calcification in patients with ESRD. In addition, 25(OH)D reflects the level of vitamin D in the body, and a decrease in that can promote vascular medial calcification and contribute to the high mortality in patients with ESRD (Metzger et al., 2013). Accordingly, our study further demonstrated that renal transplantation could improve Ca and P metabolism disorders in ESRD patients. Therefore, reducing the high level of Ca and P load as well as reasonable supplement of vitamin D before renal transplantation can help to reduce vascular medial calcification.

Exosomes are an extracellular vesicle that can be produced by various types of cells and serve as an important participant in intercellular communication by transporting a variety of bioactive materials to other cells under physiological and pathological conditions (Li F. X. et al., 2019; Lin et al., 2019b). Recently, extracellular vesicle-induced vascular medial calcification was one of the mechanisms of accelerating vascular calcification in patients with CKD (Viegas et al., 2018). Accordingly, our results showed that the protein concentrations in ESRD-Ex were higher than that in Nor-Ex. Besides, the expression of Runx2 and mineralized nodules was increased significantly in VSMCs treated with ESRD-Ex. These data suggested that plasma exosomes were involved in the regulation of vascular calcification.

Increasing evidence had shown that MGP, Fetuin-A, Rankl, BMP2, Annexin-A2, and other molecules were related to vascular calcification, and the level of calcification promoters was often increased and accompanied by a decrease in calcification inhibitors in patients with CKD (Chen et al., 2008; Marechal et al., 2011; Viegas et al., 2015; Roumeliotis et al., 2020). MGP is an important calcification inhibitor, and it was significantly downregulated in the calcified arteries (Viegas et al., 2015). Recent studies further proved that high levels of inactive MGP, also known as uncarboxylated MGP (ucMGP), were significantly correlated with vascular medial calcification in patients with CKD (Schurgers et al., 2013; Viegas et al., 2015). At the same time, Thamratnopkoon et al. showed that the level of ucMGP was increased with the progression of CKD (Thamratnopkoon et al., 2017). However, the concentration of ucMGP depends on the nutritional status of human vitamin K (Viegas et al., 2015; Nollet et al., 2019). In order to avoid the effects of vitamin K, the total content of MGP, including ucMGP and carboxylated MGP, was detected in our present study, and the results showed that both the level of plasma and plasma exosomes total MGP were much lower in ESRD and RTR patients compared with that in normal health control. Fetuin-A is another key calcification inhibitor, and it inhibits the formation and precipitation of apatite precursors by enhancing the expression of MGP (Holt and Smith, 2016). Many studies have confirmed that low levels of Fetuin-A are related to vascular calcification, and it is an independent risk factor for cardiovascular events in patients with ESRD (Marechal et al., 2011; Scialla et al., 2014; Chen et al., 2020). Consistent with these previous research results, our present study showed that the level of Fetuin-A and total MGP were much lower in both plasma and plasma exosomes of ESRD and RTR patients.

In addition, elevated level of serum BMP-2 in patients with CKD-induced VSMCs calcification and Rankl could promote vascular calcification by inducing the release of BMP-2 by vascular endothelial cells (Chen et al., 2006; Davenport et al., 2016). Besides, Annexin-A2 could bind to Fetuin-A at the cell membrane of VSMCs in the presence of high calcium and contribute to SMCs and osteoblast-derived matrix vesicle-mediated calcification (Chen et al., 2008; New et al., 2013). Accordingly, our results demonstrated that the content of Annexin-A2 was much higher in ESRD-Ex and RTR-Ex than that in Nor-Ex, but that of Rankl and BMP-2 had no difference among the three groups. On the other hand, the plasma concentration of Annexin-A2 and BMP-2 were increased significantly in patients with ESRD when compared with normal health control. However, the plasma concentration of Rankl was a little lower in patients with ESRD, but there was no significant difference among the three groups. CAC is highly prevalent and severe in patients with ESRD, and it independently predicts the risk of total cardiovascular diseases (Chen et al., 2017). Interestingly, we found that the content of both Fetuin-A and MGP in plasma exosomes was negatively while that of Annexin-A2 was positively related to CACS. Therefore, our experiment demonstrated that the lower levels of MGP and Fetuin-A, but high level of Annexin-A2, might be one of the reasons that ESRD-Ex promote VSMCs calcification.

However, there are important limitations of this study that are worth mentioning. First, only eight subjects were included in each group, and it is necessary to expand the samples to enhance the power of our data analyses. Second, the origin of exosomes and the bioactive materials carried by exosomes to regulate VSMCs calcification need further study, and it is not fully understood whether other substance in plasma are involved in regulating vascular calcification. Third, the detailed relationship between the concentrations of calcification-related factors in plasma and plasma exosomes need further study, and the molecular mechanism of modulating the levels of calcification-related factors (MGP, Fetuin-A, and Annexin-A2) was not clarified in this study. Last but not the least, it is important to apply exosomes to animal model and follow up further on the situation of vascular calcification in RTR patients.

In summary, our results demonstrate that the disorders of Ca and P metabolism and the contents of plasma exosomes are important factors that trigger vascular calcification in ESRD patients. Moreover, the decrease in calcification inhibitors and the increase in some calcification promotors in both plasma and plasma exosomes play a key role in the process of vascular calcification in patients with ESRD. Interestingly, renal transplantation could partially attenuate vascular calcification. Therefore, the present study provides new insights for preventing and treating vascular calcification and hope for improving the quality of life and reducing medical costs for patients with ESRD.

## Materials and Methods

### Patients and Specimens

A total of eight patients with CKD5 in the Department of Nephrology and eight patients who underwent post-operative renal transplantation re-examination in the Department of the Center of Organ Transplantation during the same period were selected at the Second Xiangya Hospital, Central South University. Meanwhile, eight normal healthy age paired volunteers in the physical examination center were recruited in this study. They were divided into three groups: ESRD, RTR, and normal healthy control (Nor). The inclusion criteria were as follows: CKD patients with the glomerular filtration rate (GFR) <15 ml/min/1.73 m^2^ of body-surface areas were selected as the ESRD group. Patients with renal transplantation due to ESRD but whose renal function was normal were enrolled into the RTR group. The healthy volunteers with normal renal function and without other diseases such as a malignant tumor, hypertension, diabetes, coronary heart disease, and so on were enrolled into the Nor group. The clinical parameters of all the participants in this study are presented in Table 1.

### Coronary Artery Calcification Measurement

CACS were calculated using the Siemens Somatom Definition computed tomography (CT) multilayer spiral scanner (Germany), and the calcification of coronary arteries was quantified via Agaston and analyzed by Siemens CaScoring software (syngo. via, Siemens Healthcare GmbH). The width of the detector was 128 × 0.6 mm, and the scan thickness was 1.5 mm simultaneously over 50–70 images of fault scans. A total coronary artery calcification score was generated by using the Agatston method, which has been described before (Bashir et al., 2015). The vessels evaluated included the left main coronary artery (LM), left anterior descending coronary artery (LAD), left circumflex (CX), and the right coronary artery (RCA). The measure of the area of calcification lesions times a fixed coefficient (the maximum pixel density decision) and the total score of the calcification of all faults was termed as the CACS.

### Exosomes Extraction

Blood was obtained from patients with ESRD, RTR, and Nor, respectively. The blood was centrifuged at 3,000 × g for 20 min, and the supernatant plasma was subjected to the ExoQuick-TC Exosome Precipitation Solution Kit (System Biosciences, USA) for exosomes extraction. Briefly, 250 μl of plasma was added to 63 μl ExoQuick Exosomes Precipitation Reagent and fully mixed. After the mixture had been incubated at 4°C for 30 min, it was centrifuged at 1,500 × g for 30 min at room temperature (RT), and the yellow or white precipitate at the bottom of the EP tube was the exosomes. Then, the supernatant was removed by aspiration, and the exosomes pellets were centrifuged again at 1,500 × g for 5 min. Finally, the exosomes pellets were resuspended in 200 μl phosphate-buffered saline (PBS). The protein content of exosomes was determined by the bicinchoninic acid (BCA) kit (Beyotime Biotechnology, Shanghai, China).

### Identification of Exosomes

The morphology of plasma exosomes was detected by TEM. Briefly, exosomes were fixed with equal volumes of 1% phosphotungstic acid (pH 7.4). A 10 μl sample was loaded onto a bronze net with film after washing and holding at RT for 10 min. Then, 10 μl of phosphotungstic acid staining solution was added to the negative stain, and it was observed under a Hitachi H-7650 TEM (Hitachi, Tokyo, Japan). A particle and molecular size analyzer (Zetasizer Nano ZS; Malvern Instruments) was used to measure the size distribution of the exosomes. The expression of exosome surface markers (CD9, CD63, and CD81) were analyzed by western blot analyses.

### VSMCs Uptake Exosomes

VSMCs were isolated from 6- to 8-week-old C57/BL male mice as described before (Lin et al., 2019a). VSMCs were cultured in Dulbecco's modified Eagle's medium (DMEM) supplemented with 10% fetal bovine serum (FBS) (Gibco BRL Co. USA), penicillin (100 U/ml), and streptomycin (100 μg/ml) at 37°C in a humidified atmosphere of 5% CO_2_. VSMCs were treated with 100 μg of ESRD-Ex, RTR-Ex, and Nor-Exo, and the calcification levels were evaluated. Exosomes were labeled with PKH26 (MINI26, Sigma, USA) according to the manufacturer's instructions. Briefly, 4 μl of PKH26 fluorescent solution was dissolved in 1 ml of diluting solution C, and then, the mixed solution was added to 40 μl exosomes. After stopping the reaction by using 5% bovine serum albumin (BSA), the mixture was ultracentrifuged at 100,000 × g for 70 min, and the labeled exosomes were incubated with VSMCs at 37°C for 12 h. After fixing the cells with 4% paraformaldehyde for 30 min at RT and washed three times with PBS, 4′,6-diamidino-2-phenylindole (DAPI) (Invitrogen, Carlsbad, USA) was added for 5 min. After washing with PBS three times, the staining signals were analyzed with a fluorescence microscope (DMI6000B, Leica, Germany).

### Alizarin Red S Staining

VSMCs were coincubated with β-glycerophosphate (β-GP, 10 mmol/L) and ESRD-Ex, RTR-Ex, or Nor-Ex (100 μg/ml), respectively, for 21 days, and the Alizarin Red S staining was done as before (Lin et al., 2019a). The cells were fixed and then stained with 1% (pH 4.2) Alizarin Red S for about 10 min. The mineralized nodules were assessed and photographed with a microscope. To quantify calcium levels, the cells were washed with PBS and decalcified with 0.6 N HCl for 24 h. Calcium content was determined by measuring the concentration of calcium in the HCl supernatant by atomic absorption spectroscopy. After decalcification, the cells were washed with PBS and solubilized with 0.1 N NaOH/0.1% sodium dodecyl sulfate (SDS). The protein content was measured with a BCA protein assay (Beyotime Biotechnology, Shanghai, China). The calcium content was normalized to the protein content.

### Western Blot Analyses

The expression of proteins was determined by western blot as previously described (Lin et al., 2018). Briefly, the concentration of protein was detected using a BCA kit (Beyotime, Shanghai, China). 30 μg total protein was loaded onto 8% or 12% SDS-PAGE gel and transferred to polyvinylidene fluoride (PVDF) membranes. The membranes were incubated with primary antibody at 4°C for over 12 h after blocking with 5% non-fat milk for 1 h. Subsequently, horseradish peroxidase (HRP)-conjugated goat–anti-rabbit (sc-2004, 1:5,000, Santa Cruz) or HRP-conjugated goat–anti-mouse (sc-2005, 1:5,000, Santa Cruz) secondary antibodies were used to incubate with the membrane at RT for 1 h. The immunoreactive bands were visualized using the ECL Plus Western Blot Detection Kit (Amersham Biosciences U.K. Ltd.). The relative expression levels of proteins were normalized to the intensity of the β-actin band. Primary antibodies including CD9 (ab92726, 1:1,000, Abcam), CD63 (ab68418, 1:1,000, Abcam), CD81 (ab79559, 1:1,000, Abcam), Runx2 (ab76956; 1:1,000, Abcam), Fetuin-A (16571-1-AP, 1:1,000, Proteintech), MGP (10734-1-AP, 1:1,000, Proteintech), Annexin-A2 (11256-1-AP, 1:1,000, Proteintech), BMP-2 (ab214821, 1:1,000, Abcam), Rankl (23408-1-AP, 1:500, Proteintech), and β-actin (ab6276, 1:3,000, Abcam).

### ELISA

The contents of Fetuin-A (ab108855), MGP (CSB-E09714h), Annexin-A2 (CSB-E12156h), BMP-2 (CSB-E04507h), and Rankl (ab213841) in both the plasma and plasma exosomes were detected using an ELISA kit according to the manufacturer's instructions. Briefly, 0.1 ml sample or standard was added into each hole of the reaction plate and incubated at 37°C for 2 h. After discarding the liquid, 0.1 ml of biotin labeled antibody working liquid was added and put on the new plate paste, continuing to incubate at 37°C for another 30 min. Then, the liquid was discarded again and washed three times. One hundred microliters working liquid was added, and it was covered with the new post and incubated at 37°C for 1 h. The liquid was dropped and washed five times. Finally, 90 μl of substrate solution was added to each hole, and coloration was done at 37°C for 15–30 min away from light. Then, the optical density (OD) value of each hole was measured with a microplate at the wavelength of 450 nm within 5 min after termination of the reaction.

### Statistical Analysis

The data were presented as means ± standard deviation (SD), and they were analyzed with GraphPad Prism software (GraphPad Prism version 6.0). The Student's *t*-test was used to compare normally distributed data between two different groups, while one-way analysis of variance (ANOVA) together with a Tukey's *post-hoc* test was used for multiple groups. A level of *p* < 0.05 was considered statistically significant. All experiments were repeated at least three times, and representative images were shown in the figures.

## Data Availability Statement

The original contributions presented in the study are included in the article/Supplementary Materials, further inquiries can be directed to the corresponding author/s.

## Ethics Statement

The studies involving human participants were reviewed and approved by the Ethics Committee of the Second Xiang-Ya Hospital, Central South University. The patients/participants provided their written informed consent to participate in this study.

## Author Contributions

L-QY conceived and designed the experiments. XL and TZ performed the experiments, analyzed the data, and prepared all the figures. J-YZ, FX, FL, S-KS, FW, BG, M-HZ, YW, Q-SX, X-BX, X-BL, and H-YL provided technical support, made substantial contributions to data analysis, and revised the manuscript critically for important intellectual content. XL and TZ wrote the manuscript. All authors read and approved the manuscript and agree to be accountable for all aspects of the research in ensuring that the accuracy or integrity of any part of the work are appropriately investigated and resolved.

## Conflict of Interest

The authors declare that the research was conducted in the absence of any commercial or financial relationships that could be construed as a potential conflict of interest.

## Abbreviations

CKD: chronic kidney disease
ESRD: end-stage renal disease
RTR: renal transplant recipients
Nor: normal
Ex: exosomes
ESRD-Ex: the plasma exosomes derived from ESRD patients
RTR-Ex: the plasma exosomes derived from renal transplant recipients
Nor-Ex: the plasma exosomes derived from normal health control
VSMCs: vascular smooth muscle cells
DMEM: Dulbecco's modified Eagle's medium
β-GP: β-glycerophosphate
MGP: matrix gla protein
BMP-2: bone morphogenetic protein
Rankl: receptor activator for nuclear factor-κB ligand
CAC: coronary artery calcification
CACS: coronary artery calcification scores
TG: triglyceride
TC: total cholesterol
HDL: high-density lipoprotein
LDL: low-density lipoprotein
Cre: creatinine
BUN: blood urea nitrogen
UA: uric acid
csCa: corrected serum calcium
Ca: calcium
P: phosphorus
Ca × P: calcium–phosphorus product
PTH: parathyroid hormone
ucMGP: uncarboxylated MGP
GFR: glomerular filtration rate
CT: computed tomography
TEM: transmission electron microscope
RT: room temperature.
