# Supplementary material for: Plasma Exosomes Derived From Patients With End-Stage Renal Disease and Renal Transplant Recipients Have Different Effects on Vascular Calcification
Source: Front Cell Dev Biol. 2021 Jan 28;8:618228. doi: 10.3389/fcell.2020.618228 (PMC7876285; doi:10.3389/fcell.2020.618228)
Supplement: Supplementary file 1 [file Data_Sheet_1.docx]

**Supplemental materials**

**Supplemental figure 1**


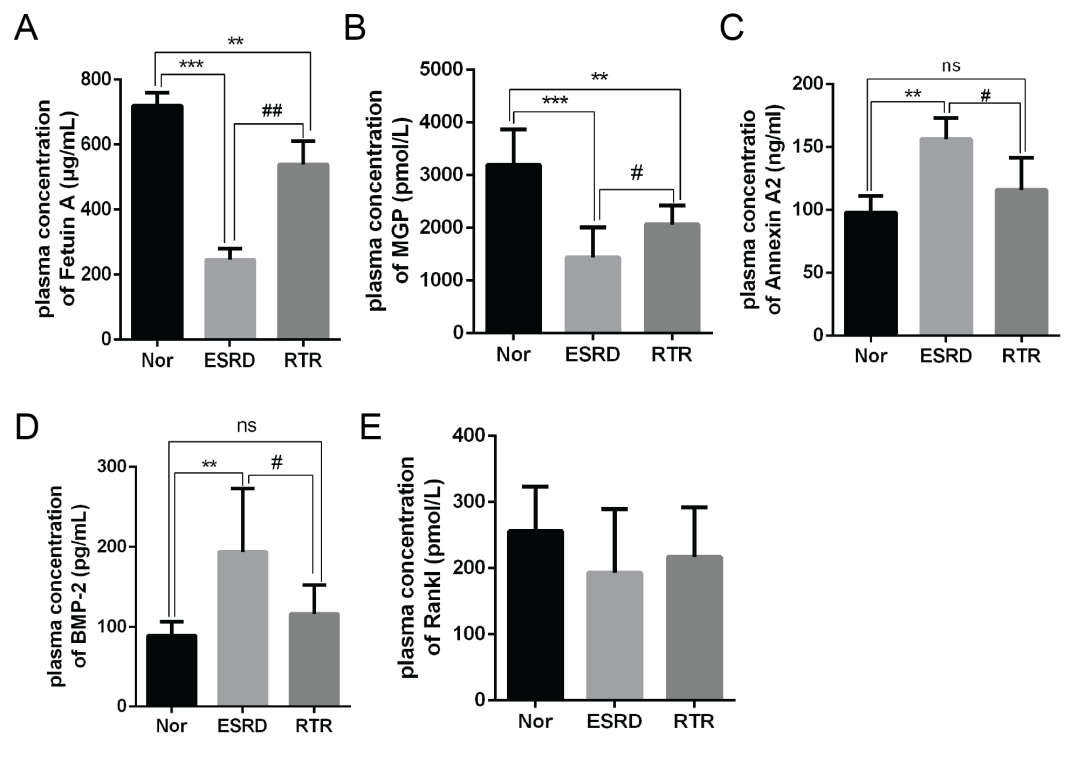


**Supplemental figure 1. The plasma concentration of calcification related factors.** (A-E) The different plasma concentration of Fetuin-A, MGP, Annexin-A2, BMP-2 and Rankl in Nor, ESRD and RTR patients were measured by ELISA. n=8. ***p<0.001, **p<0.01, *p<0.05, compared with Nor. ##p<0.01, #p<0.05, compared with ESRD. ns: no significant. Nor: normal health control; ESRD: end stage renal disease; RTR: renal transplant recipients.
